# *‘It feels it’s wasting whatever time I’ve got left’*: A qualitative study of living with treatable but not curable cancer during the COVID-19 pandemic

**DOI:** 10.1177/02692163211049497

**Published:** 2021-10-19

**Authors:** Eloise Radcliffe, Aysha Khan, David Wright, Richard Berman, Sara Demain, Claire Foster, Susan Restorick-Banks, Alison Richardson, Richard Wagland, Lynn Calman

**Affiliations:** 1 University of Southampton, Southampton, UK; 2 The Christie NHS Foundation Trust, Manchester, UK; 3 Patient Representative, Southampton, UK

**Keywords:** COVID-19, cancer, palliative, carers, qualitative

## Abstract

**Background:**

People living with cancer that is treatable but not curable have complex needs, often managing health at home, supported by those close to them. Challenges are likely to be exacerbated during the COVID-19 pandemic and the risk-reducing measures introduced in response. The impact of COVID-19 on those living with incurable, life-threatening conditions is little understood.

**Aim:**

To investigate the experiences and identify the impact of the COVID-19 pandemic for people living with treatable not curable cancer and their informal carers.

**Design:**

Qualitative semi-structured phone interviews were conducted with 21 patients living with cancer that is treatable but not curable and 14 carers.

**Setting/ participants:**

Participants were part of a larger longitudinal qualitative study (ENABLE) on supported self-management for people living with cancer that is treatable but not curable.

**Results:**

The COVID-19 pandemic magnified uncertainty and anxiety and led to loss of opportunities to do things important to patients in the limited time they have left to live. Lack of face-to-face contact with loved ones had a significant impact on patients’ and carers’ emotional wellbeing. Carers experienced increased responsibilities but less access to formal and informal support and respite. While changes to treatment led to some concern about longer-term impact on health, most patients felt well-supported by healthcare teams.

**Conclusion:**

The study provides rich insights into the nature of challenges, uncertainty and lost opportunities resulting from the COVID-19 pandemic for patients and carers living with cancer that is treatable but not curable, which has wider resonance for people living with other life-limiting conditions.

**What is already known on this topic?**

- People living with cancer that is treatable but not curable have complex needs, often managing their health and wellbeing at home, supported by those close to them.
- People living with cancer that is treatable but not curable often wish to maintain independence, normality and control over their lives.

**What this paper adds?**

- Patients living with cancer that is treatable but not curable and their carers experienced heightened uncertainty and a sense of lost opportunities as a result of the COVID-19 pandemic.
- While changes to treatment led to some concern about the longer-term impact on their health, most patients reported feeling well-supported by healthcare teams.
- Findings have wider resonance for people living with other life-limiting conditions.

**Implications for practice, theory or policy?**

- It is important that the psychosocial impact of the COVID-19 pandemic on patients and carers is recognised by those who commission and deliver cancer care.
- The health and wellbeing of carers needs to be acknowledged and further efforts made to include them in clinical consultations.

## Background

People living with treatable but not curable cancer increasingly live for months or years with incurable disease. They often undergo multiple episodes of treatment that can delay progression of cancer, reduce burden, alleviate symptoms and prolong life,^1,2^ as well as receiving support from palliative or enhanced supportive care services. Based on UK data there are at least 136,000 people living with treatable but not curable cancer in the UK, including those whose cancer has metastasised or recurred.^3^ However there is a lack of comparable international data on how many people are living with cancer that is treatable but not curable.^1^ It has been estimated that in the US there are at least 300,000 living with advanced cancer, although no US database exists on people living with advanced disease.^4^ UK data suggests that 10s of 1000s of people living with treatable but not curable cancer have unmet needs and are more likely to experience fear or anxiety and to require support with pain, sleep problems and fatigue compared to the general cancer population.^3^ People with treatable but not curable cancer have complex needs, mainly managing their health and wellbeing at home, supported by those close to them, and often wish to maintain independence, normality and control over their lives.^5–8^

These challenges are likely to be exacerbated during the pandemic caused by severe acute respiratory syndrome coronavirus 2 (SARS-CoV-2), the causative virus of coronavirus disease (COVID-19), identified in December 2019. People contracting COVID-19 may be at risk of severe events requiring admission to intensive care and invasive ventilation, with people with cancer at greater risk.^9,10^ Measures including social distancing and shielding, were recommended to minimise COVID-19 exposure for those deemed clinically extremely vulnerable, including those with cancer. Cancer services rapidly adapted with deferral of treatments, changes to treatment regimens, virtual consultations and remote surveillance.^11–13^ Specialist palliative care services took a flexible, adaptive approach, increasing outreach services and using digital technology to facilitate communication.^14^

The COVID-19 pandemic and resulting risk-reducing measures introduced may have a detrimental impact on the psychosocial wellbeing of people with cancer. Fear of infection and disruption to care can heighten anxiety, and isolation can undermine psychological health.^12,13,15^ A recent large UK survey of people living with cancer estimated more than 650,000 (22%) experienced disruption to treatment or follow up care due to COVID-19. For around 150,000 people this included delayed, rescheduled or cancelled treatment, with over half worried this could affect their chance of survival.^16^ Similarly other UK surveys of people with cancer found a high level of reported disruption to cancer care and a negative impact on emotional well-being^17–19^ and a large survey in Singapore found over half of people with cancer and nearly three quarters of their carers feared COVID-19 may affect their cancer treatment.^20^ However, the full psychosocial impact of COVID-19 and risk-reducing measures including social restrictions, on those living with incurable, life-threatening conditions and their carers, who often live in the same household, is little understood. Our study addresses this gap by focusing on the following objectives:

1. Investigate the experiences of people living with treatable but not curable cancer and their carers during the COVID-19 pandemic
2. Identify the impact of the COVID-19 pandemic on people living with treatable but not curable cancer and their carers
3. Make recommendations about how services can be adapted to address the impact of COVID-19 on people living with treatable but not curable cancer and their carers

## Methods

This was a qualitative exploratory interview study, reported following the Consolidated Criteria for Reporting Qualitative Research guidelines.^21^

### Participants

This study was embedded within a larger longitudinal qualitative study (ENABLE) on supported self-management for people with treatable but not curable cancer. People living with treatable but not curable cancer (referred to as ‘patients’) and friends or family members who provided informal care and support (referred to as ‘carers’) were recruited from outpatient clinics at a large University Hospital Trust in the South of England and a tertiary cancer centre in the North. Recruitment involved a demographically broad sample, representing experiences of different health and other support services.

Patients with treatable but not curable cancer were purposively sampled with reference to age (16 and over), gender and cancer type,^22^ diagnosed with four types of cancer (see Table 2), to represent a range of patients. Clinical care teams identified eligible patients and introduced them to researchers (one based at each site) who provided a participant information pack and discussed the study with them (and their carer, if applicable). Patients without a carer present were asked to nominate a ‘person they get most support from’ (aged 16 and over) who may wish to participate. Those without a nominated carer were also eligible. Interested patients/carers completed a reply slip and were contacted by the researcher a few days later to arrange a face-to-face meeting. A total of 60 patients were invited to participate, 30 of whom agreed and 22 had carers who agreed to participate making a total of 52 participants. Twenty-four patients declined, mainly due to ill health and commitments of medical appointments, and six could not be contacted using the reply slip details. (See ENABLE published protocol for further details on study design, recruitment and sample^23^).

When the first UK COVID-19 lockdown and implementation of shielding guidance for clinically vulnerable people commenced in March 2020, it was decided that an embedded study should focus on participants’ experiences of living through the pandemic. Therefore for the current embedded study on the impact of COVID-19 an opportunistic sample of patients and carers participating in the ENABLE study and due for a follow-up interview were recruited. Of the 52 ENABLE participants, 35 (21 patients and 14 carers) due a follow-up interview between March and June 2020, were approached for the COVID-19 specific interviews. None declined. All participants had previously provided written consent to participate in three interviews, and all gave verbal consent to participate in an interview on the impact of COVID-19.

### Data collection

Both interviewers were employed as researchers and were experienced qualitative researchers, (ER has a PhD, AK an MPH), female and independent of the clinical setting. Interviews were conducted using an interview topic guide. The original ENABLE topic guide was piloted with support from the patient and carer User Reference Panel), and this was adapted to address experiences and impact of COVID-19 (see box 1), in consultation with the patient representative who chairs the User Reference Panel (SRB). Interviews were audio recorded and transcribed verbatim, and in the interests of time were not returned to participants for comment.

**Box 1.**
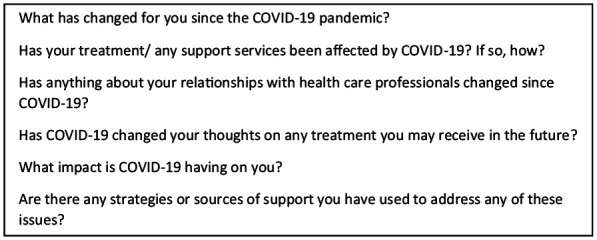
Additional interview topic guide questions on COVID-19.

Interviews covered sensitive topics with participants who were potentially vulnerable. However participants were only approached by an agreed member of their local clinical team and the research team have much experience in conducting research with people living with cancer. A plan for any participants who experienced distress during the interviews included stopping or pausing the interview if necessary, and providing details of resources for further support.

ENABLE received ethical approval from the UK Health Research Authority (South Central – Hampshire A Research Ethics Committee reference 19/SC/0132 on 29.4.19).

ENABLE interviews commenced as face-to-face, however due to COVID-19, these were replaced with phone interviews. All patients and carers were interviewed separately, except for one patient and carer who requested to be interviewed together. Interviews lasted an average of 35 minutes. No fieldnotes were taken but basic demographic information was recorded (see Table 1).

**Table 1.** Participant sociodemographic characteristics.

	Patients (*n* = 21)	Carers (*n* = 14)
**Age years (%)**		
40–50	0 (0)	2 (14.3)
51–60	6 (28.6)	2 (14.3)
61–70	8 (38.1)	5 (35.7)
71–80	6 (28.6)	5 (35.7)
**Gender**		
Male	17 (81)	2 (14.3)
Female	4 (19.0)	12 (85.7)
**Ethnicity**		
White British	20 (95.2)	12 (85.7)
British Indian	1 (4.8)	0 (0)
White other	0 (0)	2 (14.3)
**Living arrangements**		
Living with spouse	14 (61.9)	12 (78.6)
Living alone	5 (23.8)	1 (7.1)
Living with spouse and dependent children	1 (9.5)	1 (14.3)
Living with dependent child, no spouse	1 (4.8)	0 (0)
**Employment status**		
Working	3 (14.3)	3 (21.4)
Not working due to COVID-19	2 (9.5)	2 (14.3)
Not working due to ill health	2 (9.5)	0 (0)
Retired	14 (66.7)	6 (42.9)
Stopped working due to caring role	N/A	3 (21.4)

### Analysis

Interview data were analysed thematically, drawing on elements of Rapid Analysis methods designed to deliver timely findings with methodological rigour, using a deductive, explanatory approach,^24,25^ but allowing flexibility to ensure the full range of experiences were captured. The two researchers read and re-read all interview transcripts, recording key themes and general reflections in a summary table. Key themes were developed into a detailed coding framework, applied through the systematic coding of 12 interviews (six interviews coded independently by each researcher). Any discrepancies were resolved in collaboration with other research team members. The two researchers then iteratively refined and added to the coding framework once data saturation occurred. The framework was applied to the remaining interviews, using QSR NVivo 12 for data management.

Feedback on preliminary findings was provided by a group of seven clinicians who met face-to face, ensuring clarity and drawing out considerations for practice. A consultation over video call then followed with the User Reference Panel, chaired by the patient representative (SRB), to provide reflection and verification of findings.

## Results

### Participants

Thirty-five participants (21 patients and 14 carers) took part in a COVID-19 specific interview (see Tables 1 and 2 for characteristics).

**Table 2.** Patient characteristics (*n* = 21).

**Cancer type (%)**	
Myeloma	10 (47.6)
Prostate	7 (33.3)
Gynae	2 (9.5)
Gastrointestinal	2 (9.5)
**Treatment type**	
Active treatment	15 (71.4)
Not on active treatment	6 (28.6)
**Years post-diagnosis (years)**	
1–5	13 (61.9)
6–10	6 (28.6)
11–15	2 (9.5)
**Average years post-diagnosis (years)**	5.24

Patients’ level of mobility varied: most were able to walk unaided and carry out self-care activities independently; a small number of patients required a walking aid and assistance with self-care activities. Some were experiencing relatively stable health at the time of interview, whilst others health had deteriorated since the previous interview.

Of the 14 carers, 13 were spouses and lived with the patient; one cared for his father. The carers were slightly younger than patients and generally in good health with high levels of mobility.

Below are the main findings, supported by participants’ quotes, using pseudonyms. For ease of reading some text has been removed in a number of quotes (indicated by ‘. . .’)

### Impact on health and psycho-social wellbeing

The COVID-19 pandemic had magnified feelings of uncertainty and anxiety already experienced by patients with treatable but not curable cancer and their carers, particularly in relation to how long they had left to live. Patients and carers expressed fears and anxieties about COVID-19, expressing concern that it presented greater risks to their health in comparison to the general population. Patients’ wishes for independence, normality and control over their lives were greatly impeded by the pandemic and the social distancing and shielding measures they were living under, with no certain end-date at the time of interview.

In general, participants felt a sense of loss at being unable to do the things that were important to them in the time they had left to live, including spending time with loved ones and participating in leisure activities:

*It feels that it’s wasting whatever time I’ve got left. . .If I get to 80 I’ll think I’ve done alright. I have this feeling of frustration that what I want to do between now and turning 80 I’m not going to be able to do it because of COVID-19, not because I’m not fit to do it. I want to go abroad to play golf again with my chums, with my family. I want to be able to regularly visit the grandkids, go to the pub with my mates, go and play golf. Will I ever be able to do these things again with this COVID? Who knows?*

(Cedric, aged 70s, prostate cancer patient, lives alone)

The impact of not being able to see loved ones was profound. In previous interviews, patients had emphasised the importance of friends and family, and since their cancer diagnosis there was a sense of prioritising spending time with loved ones, children and grandchildren in particular. Although not all patients and carers had internet access, most increasingly used mobile phones, video calls and social media to stay in touch with friends and family. Some had pursued new activities including online quizzes and games with their family:

*It’s very, very annoying that I can’t go and see my family. That’s what I do miss, going to see my grandchildren. . . We have a quiz (online), one of us sets it all up and we’ve even played Bingo. . . our daughter organises it, she bought the books and everything else and gave us the App. . . we have a laugh and a joke and it’s very good. . . So we’re all sat round our screens later on at 7.30pm and we have a quiz. That’s our little family.*

(Fred, aged 70s, myeloma patient, lives with his wife)

Patients and carers could no longer enjoy the social activities that were important in keeping them active and maintaining a sense of purpose, including voluntary work, social gatherings and exercise such as golf and walking. For some patients, aspects of independence had been taken from them by the pandemic. For example, Ethel took pride and pleasure in buying and preparing her own food, but now had to rely others;

*You need to keep eating properly. It’s a shame because I can’t get out to do my own shopping. . . Beggars can’t be choosers. . .That’s the trouble when somebody else is doing your shopping you don’t know what you are going to expect. . . I like to pick up my own stuff. That’s the pleasure of making your meal isn’t it, having nice vegetables. People say why don’t you have these ready-made meals being delivered and all that. I have tried them before but I don’t like them at all.*

(Ethel, aged 70s, myeloma patient, lives alone)

Some patients and carers had to cancel leisure travel plans due to COVID-19. In previous interviews they spoke about the value of planning trips away between treatments as something to look forward to, and a coping mechanism. Although some patients felt they would have future opportunities for leisure travel, others felt this unlikely due to their deteriorating health.

### Impact on health and social care provision

Although no patient’s cancer treatment was stopped due to COVID-19, some had their treatment regimen altered or were receiving treatment at a different hospital. While changes to treatment led to some concern about longer-term impact on health, most patients and carers reported feeling supported by and having trust in their clinical care teams, in particular their consultants and Clinical Nurse Specialists:

*I’ve always said I trust the professionals. I would still want to perhaps sit down with (my) Dr. . .next time I’ll probably ask [him] long term. . . is it detrimental to my maintenance. . ..I trust the NHS and also I can totally see the logic behind trying not to get people in there, which I think I totally agree with.*

(Sam, aged 60s, myeloma patient, lives with his wife)

*Whether there’s a second wave or future risk and we’ll just review it and we appreciate that there’s that risk versus his active treatment as and when but I know they’re a good health team at the hospital. . . they really are good and they’re excellent actually and so those decisions, as we go forward, I know will be between my husband and the team.*

(Olivia, aged 50s, carer, lives with husband)

At the time of the interview all patients were having phone consultations with their clinical care team instead of face-to-face appointments. As patients had established relationships with their clinical care team, they were generally very accepting of phone consultations, not wanting to put themselves at greater risk by visiting the hospital more than necessary. Many patients and carers spoke about the advantages of phone consultations, mainly the absence of travel and long waiting times.

Most patients only left home to receive treatment in hospital, with some expressing concern regarding the risks involved but feeling it was essential. However, for appointments considered ‘non-essential’, such as scans and blood tests, some patients had made difficult decisions to cancel them after weighing up the risks:

*The treatment, it’s a bit annoying at present because when I last went to the hospital and they arranged for my scan. I then got a letter through saying that my next appointments would be at the nurse led clinic, that’s not in the hospital it’s in the middle of town. I have cancelled that because of the COVID because I didn’t want to go into a place where there are. . .people who could possibly have the COVID.*

(Dave, aged 70s, Prostate cancer patient, lived with wife)

Patients could no longer be accompanied by carers or family members while attending hospital. Although not viewed as problematic for most, some found it challenging if they relied on their carers for physical or emotional support.

Some patients and carers spoke about missing the support and social interaction provided by other services they had previously used, such as hospices, services run by charities (such as Macmillan Cancer support) and peer support groups, although some services had provided phone support:

*I used to go into the Macmillan [cancer support] centre and just have a little chat and they always make you a cup of tea before your appointment or something like that. I used to like going in. . ..There are different things going on in there too, sometimes they do yoga sitting in a chair. . . That’s not happening at the moment, no. I just have to stick it out.*

(Ethel, aged 70s, myeloma patient, lives alone)

### Impact on carers’ health and psychosocial wellbeing

Carers reported experiencing more uncertainty and anxiety in relation to the pandemic with some very concerned about the impact on patient’s mental health and wellbeing. A few talked about wanting to ensure certain affairs were in order, such as finances and wills:

*COVID-19 has taken over what is going on with (my husband’s) chemo. . . he’s high risk and I try not to think about it too much because if you think about it too much it’s too scary. . . we’d already set in motion about having somebody to redo our wills but of course they now can’t come because of (COVID-19). But they’ve also said to everybody, people should write down what their wishes are, if they contract this virus. . . So we have had those sorts of conversations. I think (my husband) does think that he won’t live long. . .Most the time he is really positive but I . . . think he feels that if he should get [COVID-19] well that would be it anyway. He said to me I won’t be able to fight it.*

(Tina, aged 50s, wife and carer)

Some carers were concerned about the health risk they posed to the patient, and that there would be no one to care for the patient if they became ill with COVID-19. Although some were shielding with the patient and not leaving home at all, most had to go out to buy groceries and collect medicines, and they talked about the importance of social distancing and hygiene. However, family, friends and neighbours also provided help with grocery shopping:

*If I get [COVID-19], well I don’t know. I think I’m more likely to recover from it than (my Dad) if we both get it. I do not want to be responsible for passing on a germ to my Dad that would finish him off. . .Because there on after to live with that for the rest of my life is, you know, that is not something that I want on my conscience at all. So I am paranoid. But I’m having to go out because my Dad, he can’t leave the house so I’m having to do the shopping and pick up the drugs.*

(Daniel, aged 40s, son and carer)

The practical challenge of following shielding advice for patients who lived with others was discussed by some carers. For example, it was often not possible for household members to use separate bathrooms and bedrooms. Some felt that shielding information should have been communicated directly to all household members in addition to patients:

*Because of the COVID-19 situation where medical personnel are. . .looking at lists with people like my husband on those lists and saying we need to inform these patients but really in a situation like this when we’re all supposed to stay at home together, they need to be informing whole households of their own expectations. . . Some of the things they were saying to [my husband] in this letter was pretty well impossible, that whole move to a separate bedroom and use a separate bathroom and that’s not. . .an option.*

(Emma, aged 40s, wife and carer)

Some carers were having to provide a greater level of practical care to patients due to the pandemic but had less access to services and support previously available, including paid carers and respite services from hospices. Shielding meant some carers also received less practical and emotional support previously provided by friends and family outside of the home, leaving some feeling isolated:

*At the time [the agency care staff] had no mask, they’d no protective gear or anything so I said I don’t think it’s a good [idea]. I’m not saying that I don’t want your help. . .We decided to stop them and I just carry on doing the caring. . . I just feel very lonely and isolated really. I mean I obviously speak to people on the phone and all the rest of it but it’s not the same is it as actually physically seeing somebody.*

(Mary, aged 50s, wife and carer)

### Adjusting to a ‘new normal’

Many patients and carers identified strategies that helped them adjust, manage, and cope with the various practical and emotional impacts of the pandemic. Many drew on previous experience of managing potential health threats, for example social distancing after treatment when their immunity was low. Some discussed becoming accustomed to staying at home within the context of their deteriorating health and increasing age. Most were retired and spoke about having time on their hands whilst at home. Maintaining a routine, completing over-due household and garden chores was often identified as a coping strategy:

*[We’re] trying to keep in a routine. . . We’re still getting up and getting ready and doing that but I’m just having to make things last. Whereas normally. . .you are charging about doing this, that and the other so I’ve been making things last. Instead of probably doing my washing and then my ironing on the same day I’m leaving my ironing so I’ve got my ironing to do the next day so that passes on another hour.*

(Mary, aged 50s, wife and carer)

A few participants discussed general positive aspects of life under lockdown, such as a slower, calmer pace of life. Some talked about lockdown as a collective nationwide experience, which made them feel less negative about missing out on holidays and other activities due to ill health or caring responsibilities:

*In a stupid sort of way it’s done me a good thing this corona[virus] because of the fact that it’s given me time to get my head sorted. . . it’s actually helped me, yes, which is a stupid thing but again you know damn well that it’s not just me in this boat, it’s the whole country and most of the world isn’t it. In a way it takes some of the responsibility and blame off me*.

(Dave, aged 70s, prostate cancer patient, lived with wife).

*I’ve felt so trapped [since my husband became ill]. . . the fact that it all went into lockdown made me feel better in a way because it just made me feel like everybody else. . . I’m not sat watching everybody else go off on holiday and I know that’s a totally selfish thing to say but that’s just how it made me feel. . . I just thought it’s been like this for me for a long time really because I feel like a single parent. . . because whatever I do I have to put my husband first and think about is he going to be OK.*

(Rita, aged 60s, wife and carer)

## Discussion

Uncertainty and anxiety about the future is a particular challenge for people living with cancer^26–28^ but they can develop coping strategies,^29^ including maintaining a sense of normality and control.^5–8^ Our research has shown the COVID-19 pandemic has magnified uncertainty, disrupted a sense of normality and control and led to loss of opportunities to do things important to patients in the limited time they have left to live. This is supported by findings from two recent qualitative studies on experiences of COVID-19 for patients living with advanced lung cancer in the US^30^ and patients living with cancer and carers in Singapore.^31^ Our findings indicated that lack of face-to-face contact with loved ones had a significant impact on patients’ and carers’ emotional wellbeing. Participants increasingly used technology to stay in contact with family and friends, as reported in the US study,^30^ however not all participants had access to, or were able to, use technology. Our carers experienced increased responsibilities during the pandemic but with less access to formal and informal social support and respite. This reflects findings that carers in Singapore reported a reinforced sense of duty towards patients^31^ and UK survey findings that carers of people with cancer reported a reduced quality of life and higher levels of anxiety, stress and depression during the pandemic.^32^

### Strengths and limitations

Recruitment to ENABLE was reliant on clinical care teams acting as gatekeepers. Despite efforts to recruit from diverse ethnic and cultural backgrounds, our sample largely comprised participants identifying as White British, although from different geographical areas of the UK, and the majority were retired. This limits wider applicability of our findings. Patients and carers were mainly in spousal relationships, reflecting the general make-up of caregiving relationships in the UK, but we acknowledge findings may have limited applicability to those in other caregiving relationships. However, we argue our findings are relevant for people living with and beyond cancer and other life-limiting conditions, providing detailed insights into the impact of COVID-19. Using longitudinal qualitative methods provided a unique opportunity to adapt the methods to phone interviews as a strong rapport had been established with participants through recruitment in person and at least one prior interview. This rapport contributed to the richness of our data. We recognise that interviews provide a snapshot of one time-point during the first UK lockdown when shielding was recommended for clinically vulnerable people, and the situation with the pandemic has been very fluid and fast-moving, with guidance from governments changing frequently in response. However, many themes that have emerged are likely to have a profound impact on patients and carers for months and years in the future.

### Implications

The pandemic has led to significant challenges for health services and palliative care services around the world have been overwhelmed during the pandemic and there have been calls for better recognition and integration of palliative care services.^33^ Strategies are currently being implemented to ensure the recovery of disrupted cancer services, such as the UK Cancer Recovery Strategy which not only focuses on capacity of diagnostic and treatment services but patients’ confidence in being cared for in a safe environment and re-establishing follow up care.^34^ Our findings indicate that patients felt well supported by their clinical care teams and were generally very positive about phone consultations, suggesting that this is an acceptable approach to health care delivery beyond the pandemic. Despite significant gaps in face-to-face support provided by health services and the third sector, there has been a rapid response by services to address this and many new and innovative resources have been developed. Our findings indicate that participants were seeking new ways of connecting and receiving support and it is recommended that support to access and use new technologies is provided where necessary. Elsewhere, based on our findings, we have suggested online and phone resources intended as a guide for health care professionals to signpost to patients and carers, including practical and emotional support, help with using technology, and resources specifically aimed at carers.^35^ However we would also encourage development of safe areas where patients and carers can meet in person with healthcare professionals and other people living with cancer, particularly for those without access to online resources or support, or when critical discussions are required and remote consultations are not appropriate.

Findings indicated some participants had concerns about the longer-term impact of changes to aspects of treatment and care. We acknowledge that healthcare professionals also have concerns about care provision and conversations within and between the professions and with the public are recommended to acknowledge the impact on clinicians and patients. There should continue to be a reflection and considered response to the long-term challenges to cancer care during COVID-19. We also recommend professionals are provided with the opportunity to prepare for difficult and complex conversations about COVID-19 and cancer. Healthcare professionals are skilled at communicating significant news and complex treatment information to patients and their families. The focus of these conversations are likely to change over time and reflect the concerns of the participants relating to the long-term impact of treatment decisions.

Based on the findings we recommend that the experiences of those shielding are fed into any new pandemic guidance, particularly in relation to patients’ contact with carers they live with. Healthcare professionals may wish to consider the role of carers and other family members who live with patients, when communicating with patients about treatment, COVID-19 and shielding. Carers may not be able to attend treatment or appointments in person and involvement in remote conversations may be difficult. It is recommended that the impact on carers’ health and wellbeing is acknowledged and further efforts are made to include them in consultations as and when appropriate and agreed with the patient.

## Conclusion

Strategies are currently being implemented to ensure the recovery of disrupted cancer services and we would urge that the psychosocial impact of the pandemic on patients and carers is recognised by those who commission and deliver cancer care and that services are also developed or adapted to address these needs. Numbers diagnosed with treatable but not curable cancer are predicted to increase in the future, in part linked to the COVID-19 pandemic and the delays in people being diagnosed due to late presentation of patients experiencing symptoms as well as healthcare service disruptions.^16,36^ This makes it even more imperative to focus on addressing the needs of this group, particularly within the context of the threat of a third wave of COVID-19 and continued pressure on health services. Our study provides detailed insights on the nature of the challenges, uncertainty and lost opportunities as a result of the pandemic for both patients and carers living with cancer that is treatable but not curable, which has wider resonance for people living with other life-limiting conditions.
